# Development of a Mobile App to Improve Numeracy Skills of Children With Autism Spectrum Disorder: Participatory Design and Usability Study

**DOI:** 10.2196/21471

**Published:** 2021-08-31

**Authors:** Theoneste Ntalindwa, Mathias Nduwingoma, Evariste Karangwa, Tanjir Rashid Soron, Alphonse Uworwabayeho, Annette Uwineza

**Affiliations:** 1 School of Education University of Rwanda – College of Education Kayonza Rwanda; 2 School of Inclusive and Special Needs Education University of Rwanda – College of Education Kayonza Rwanda; 3 Telepsychiatry Research and Innovation Network Ltd Dhaka Bangladesh; 4 School of Medicine and Pharmacy University of Rwanda – College of Medicine and Health Sciences Kigali Rwanda

**Keywords:** autism spectrum disorder, mobile app, learning, information and communication technologies, education, numeracy, mathematics

## Abstract

**Background:**

The use of information and communication technologies is transforming the lives of millions of people including children with autism spectrum disorder (ASD). However, the process of developing a user-friendly and effective mobile app needs to follow a complex standard protocol and culture-sensitive customization, and involves multiple sectors. This complex work becomes even more challenging when considering children with ASD in low- and middle-income countries as the users.

**Objective:**

This study aimed to design and develop a more intuitive mobile app to improve numeracy skills of children with ASD in Rwanda and evaluate the usability of the app.

**Methods:**

A participatory design approach was utilized in this study in which 40 children with ASD, 5 teachers, and 10 parents of children with ASD participated in focus group discussions (FGDs) and usability testing. A narrative literature review was performed to explore existing mobile apps and compare previous studies to design the questions for FGD and facilitate a framework for designing the app. The agile methodology was used to develop the mobile app, and the heuristics evaluation method was used to test and evaluate the usability of the initial version of the app to improve its functionalities. The interviews were recorded, transcribed, and analyzed following the guidelines of the qualitative narrative analysis (QNA) method.

**Results:**

During the FGDs the respondents shared their need for a mobile app in teaching and learning numeracy for children with ASD and pointed to possibilities of integrating the mobile app into existing curriculum. Ten themes emerged from the FGDs and exercise of developing the mobile app. The themes were related to (1) teaching and learning numeracy for children with ASD, (2) planning and development of a mobile app for a person with ASD, (3) testing a mobile app, (4) strength of the developed app against the existing ones, (5) behavioral maintenance and relapse prevention, (6) possibilities to integrate the mobile app into the existing curriculum, (7) data protection for users, (8) social implications, (9) challenges in Rwanda, and (10) focus on future.

**Conclusions:**

The community plays an important role in the planning, development, and evaluation of a mobile app for children with ASD. In this study, inputs from teachers and parents resulted in an optimally designed mobile app that can improve numeracy skills in children diagnosed with ASD to support the implementation of competency-based curriculum in Rwanda.

## Introduction

Autism spectrum disorder (ASD) is a neuro-developmental disorder with persistent deficit in (1) social communication and social interaction and (2) restricted or repetitive pattern of behavior, interest, or activities from the developmental period [1]. The prevalence of autism among children is increasing as a result of different biopsychosocial and environmental factors as well as with the change in diagnostic criteria. Initially, it was considered that globally around 1% of children have autism; however, recent data from the Centers for Disease Control and Prevention (CDC) indicated that 1 in 54 children have autism in the United States. As the number of children with autism is increasing, their need for health care and education is also accelerating. Studies have consistently documented that children with autism can learn and acquire different life and living skills with appropriate training and support. The study by Kamaruzaman and Azahari [2] documented the potential of these children to learn and develop their skills in various domains such as mathematics. The use of systematic instruction, such as task-analytic instruction, proved the possibility of children with significant cognitive disabilities learning basic mathematics [3]. However, only few teachers are interested about the nature and extent of ASD symptoms that cause challenges in social communication for these children; besides, their unique behavioral patterns and need for intensive support are less recognized [4-6].

Computer-based interventions available on mobile devices improve the learning of children with ASD even when they are used with minimal supervision [7]. Different studies have suggested that electronic devices such as iPads and smartphones installed with assistive apps are effective in teaching children with ASD when additional features such as audio and video are included in the provided content [8]. Previous studies have found that children can learn numeracy skills through digital devices with touchscreen technology [9,10]. Many children with ASD are drawn toward computers as they tend to be visual learners. For this reason, e-learning systems are a natural choice for them as both static and dynamic images as well as videos can be included as part of the learning process [11].

Rwanda is promoting the use of information and communication technologies (ICTs) at all levels through multiple initiatives that include the One Laptop Per Child Project (for those at basic education levels) and loan schemes for students (for those in higher learning institutions) [12]. These interventions have increased the number of computer users [13] and reduced the access gap in ICT in both urban and rural populations. Rwanda’s competence-based curriculum divides numeracy into different categories from level 1 primary (P1) to level 3 primary (P3) [14]. However, there are some children with ASD who have never attended the formal education system due to the stigma against them [15,16].

Previous studies by Tanner et al [17], Fairus et al [18], and Soomro and Soomro [19] revealed that mobile app technologies are crucial to improve the learning ability of individuals with ASD. The study by Rajagopal and Ying [20] revealed that mobile apps with repetitive instruction can improve learning numeracy skills for children with ASD. According to Díaz and Barco [21] and Alcantud et al [22], the development of assistive technologies for a person with ASD has increased in the last decade. However, few studies have taken place in sub-Saharan Africa and other low-income countries such as Rwanda [23]. According to De Macedo and Ulbricht [24], children with ASD show different behaviors, and hence parents and educators need to contextualize the content of the technological apps. Our previous work [5] revealed how worthy the development and tailor-made designs of mobile apps are and that these have immense potentiality to help improve the learning competencies of children with ASD in Rwandan schools. This study has been prompted by previous studies that revealed how mobile technologies enhance academic skills in different ways such as word matching or picture perception [25,26]. The introduction of mobile apps is the one of most attempted solutions to include persons with ASD in society [27], as the use of multimedia technologies can improve their writing skills and social communication.

The focus of children with ASD is compromised by the sensory onslaught in the learning environment which prevents them from paying attention when studying [28]. Assistive technologies such as virtual environments, augmented reality, and smart glasses [29,30] have been developed in recent years to improve the collaborative interactive environment in order to help children with ASD stay focused. The development of mobile apps to support individuals with ASD has been recommended by many researchers including Fraccaro et al [31] and Read et al [32]. However, mobile apps should be culture and context specific [12,33]. This can be achieved by including Indigenous features and examples of motivations available in their local context.

Different mobile apps such as iConverse [34] and SPEAK all! [35] have successfully improved the communication ability of children with ASD [36]. Studies by Kamaruzaman and Azahari [2] and Tashnim et al [37] introduced numeracy and calculation as a psychoanalysis to improve the lives of children with ASD. However, mobile apps designed for persons with ASD will be successful only when it is designed in response to zones of instructional opportunity, which include central coherence, a theory of mind, and executive function [38]. These zones of instruction outline a clear and validated structure of the specific instructional needs of the user, considering that the deficits and impairment associated with ASD impact the user’s learning style and also reduce the number of new skills memorized, so as to allow them to refer materials later to make the appropriate decision [39,40]. This information is significant in the argument for using mobile apps to teach and instruct children with ASD because it helps them to focus their attention on a particular topic/skill [38]. Khorrami et al [41] reported that there is a tendency among children with ASD to read by decoding before establishing an understanding of comprehension. Many studies have sought to teach simple mathematical skills (eg, number matching, counting) [42] by addressing the deficit in communication as a common impairment in children with ASD [43]. Rwanda is implementing the competency-based curriculum to develop skills for all children regardless of their disabilities from preprimary to upper secondary education [44]. Children with ASD are interested in using digital tools and their teachers and parents believe that the use of ICTs might improve their learning when the tools are developed by considering their sociocultural context [45,46]. However, how such a local culture–sensitive digital tool can be developed is not yet documented in Rwanda. This study aimed to fill this gap in knowledge by developing a user-friendly mobile app to improve numeracy skills in children with ASD level 3 in Rwanda.

## Methods

### Design

This participatory design and usability study was performed from November 2019 to June 2020 and intended to design a mobile app to improve numeracy skills in children with ASD. Mixed methodologies were employed in this study: (1) a participatory design approach [47] to improve the outcomes due to the context-sensitive need and a future-oriented approach to the design of technological solutions by involving workers and professionals from the field of education of children with ASD in the design; (2) a narrative literature review [48] to compare our app with existing mobile apps and previous studies. For this purpose, the agile [49] methodology, which considers customers, developers, stakeholders, and end users, was used to inspect the app elements at every stage of the development process and make adjustments according to the requirement; (3) a heuristics evaluation that used focus group and observation was utilized to test and evaluate the initial version of the app to improve its functionalities [50,51].

### Participants

The local participants were selected based on data from our previous study [5]. The international participants were randomly selected based on their responses to 5 questions posted on the Quora platform [52], which is an open public forum to exchange ideas.

The participants of this study were children with ASD, teachers, parents, and the international community. A total of 40 children with ASD (32 boys and 8 girls) participated in testing the developed app. Among these, 5 children (4 boys and 1 girl) were not enrolled in schools and thus were visited at their home with their parents. The remaining 35 children (30 boys and 5 girls) were enrolled in Autisme Rwanda [53], which is the center established in 2014 in Rwanda that cares for children with ASD. Five teachers (2 males and 3 females) were recruited from Autisme Rwanda. Parents (n=10) of children with ASD who participated in the design and evaluation of the app were recruited from the Rwanda Parent’s Initiative on Autism (RPIA) [54], which is an association of parents having children with ASD in their families in Rwanda. Within the parents’ group, there were 4 fathers and 6 mothers.

### Interview Guide and App Development

A series of open questions were used as the interview guide during focus group discussions (FGDs), which is a way to gather people from similar backgrounds or experiences to discuss a specific topic of interest (Multimedia Appendix 1). Some of these questions were posted on the Quora forum [52] (Multimedia Appendix 2) to collect data from the international community.

The design of the mobile app was guided by the Autism Spectrum Disorder Inclusion Collaboration Model [55]. The user interface is designed by following the principles of the Universal Mobile Application Accessibility and Inclusion [56] and included the following: (1) perceivable (to ensure that content is discernible by all users), (2) operable (ie, all features should be fully employable by everyone, regardless of the limitations of the user), (3) understandable (relates to the cognitive ability of the user to comprehend the meaning of the presented information), and (4) robust (ie, its content is flexible so it can be easily interpreted by an array of users).

The development of the app was accomplished in 2 steps: (1) user requirement analysis and (2) design process, as recommended by Soomro and Soomro [19].

### User Requirement Analysis

The app that will be designed for the education of children with ASD needed a set of requirements to improve the probability of successful learning [56]. We used detailed methods and developed a list of possible requirements from existing studies and participants [52,53,57]. The FGDs were conducted by involving teachers of special education and practitioners in the fields as participants while the children with ASD observed their behavior inside and outside the class.

Information on the software interaction design was gathered from experts around the world who had experience in treating and dealing with children with ASD as well as individuals with ASD who were successful in their academic careers. This involved posting questions on the Quora Digest platform [52] by one of the authors and analyzing their replies. We chose to use Quora as our data source because (1) Google Trends data show that it is increasing in popularity against other platforms available globally [58]; (2) it tries to match questions with experts and most questions are answered by users with authority on the content [59]; and (3) it also offers users the ability to edit the way a question is asked, and thus allows users to connect people with questions they feel the user could answer [60]. The information collected from this platform served as complimentary to data collected from teachers and parents. The profiles and responses from the Quora platform are shared as web links in Multimedia Appendix 1.

### Design Process

To understand the strength of ICT devices and examine the effectiveness of available apps for students with ASD, we next designed the mobile app interface that can respond to the needs of children with ASD in the current context. From the requirements analysis, we identified a set of characteristics needed for the software interaction, as explained by Mejía et al [25], and using the following equations:

*U*={*Sn*,*Cg*,*Mp*} **(1)**

*Sn*={*Sn*_1_, ..., *Sn_n_*} **(2)**

*Cg*={*Cg*_1_, ..., *Cg_n_*} **(3)**

*Mp* = {*Mp*_1_, ..., *Mp_n_*} **(4)**

where *U* represents a user (Equation 1), *Sn* represents the set of the senses (Equation 2), such as eyesight, hearing, and touch; *Cg* represents cognitive functions, such as memory and attention (Equation 3); and *Mp* represents a motor function for different parts of the body (Equation 4).

Designing a task-centered user interface for the app involved multiple processes, according to the established guidelines and as recommended by Punchoojit and Hongwarittorrn [55].

The steps in the task-centered design process were adapted and modified as necessary during the aforesaid process. We also referred to the universal mobile app accessibility and inclusion guidelines proposed by Ballantyne et al [56]. While designing the mobile app, 9 steps were followed: (1) task analysis, (2) choose the representative tasks, (3) find existing interfaces, (4) rough out the design, (5) analyze the user interaction, (6) create a prototype, (7) test design with users, (8) iterate, and (9) build the design.

### Task Analysis

To design this app, we first agreed on the target audience, as the background of users (ie, children with ASD) will help the designer to include elements in the design considering their ability.

In this process, we referred to the previous work by Díaz and Barco [21], where a software (*S*) is defined a set of functionalities (*F*) operated by the user, which also includes a user interface (*I*):

*S*={{*F*_1_, …, *F_n_*}*I*} **(5)**

In this mobile app, functionalities are defined as tasks (T) that can be done.

*S*={{*T*_1_, …, *T_n_*}*I*} **(6)**

Children with ASD level 3 [1] are the only actors in the system.

The mobile app includes a graphical user interface (*I*) that allows users to interact with the system. From Equation 6, the interface is

*I*={*Uip*_1_, …, *Uip_n_*} **(7)**

*Uip*={{*Cp*},{*C*}} **(8)**

Equations 7 and 8 show that an interface (*I*) is a set of user interface patterns (*Uip*) which is a group of graphic user interface–style *Cp* to solve a particular problem. It is also associated with specific characteristics of users (*C*).

**Figure 1:**
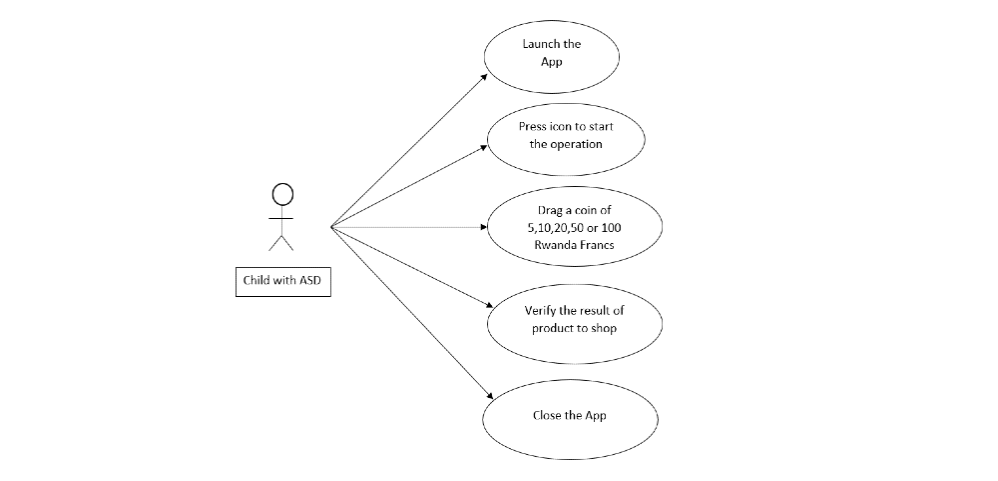
Use case diagram of the system (app). ASD: autism spectrum disorder.

The use case diagram (Figure 1) illustrates the different tasks to be performed by a user.

From Figure 1, it can be seen that each task is associated with a user interface pattern *Uip*.

∀T:*Uip* **(9)**

where ∀T represents the universal quantifier, meaning that for all cases, *T* (task) is associated with a user interface.

From Equation 9, the task includes all actions executed by the user.

*T*={*Ac*_1_, …, *Ac_n_*} **(10)**

In this app, actions are press, drag, and drop coins into a specific area.

*T*={Press, Drag, Drop} **(11)**

A child with ASD drags at least one coin of the Rwandan currency system and drops it into a provided space to get the result. Specific feedback (eg, candy, banana, strawberries, and donuts) comes immediately after a successful operation. For all users with the definite ability (A), these gifts exist as user interface patterns.

(∀*U*
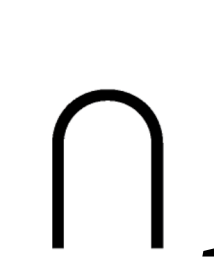
*A*)
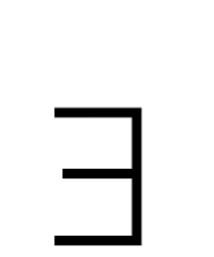
*Uip* **(12)**

The user interface pattern has coins that the user can drag and drop into the specified place, which are added up before getting a reward.

### Choose Representative Tasks

**Figure 2:**
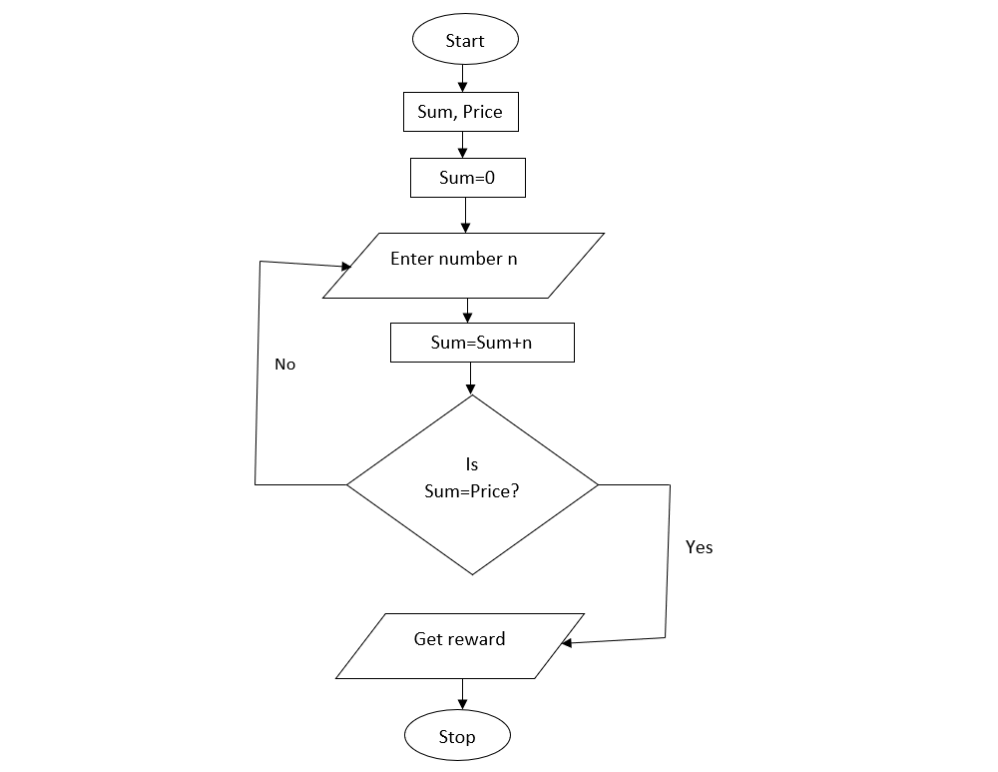
Flowchart of the developed app.

In this step, the mobile app designer analyzed a syllabus of skip counting in the competence-based curriculum [44]. Participants suggested numbers and coins to represent tasks in this app. The flowchart (Figure 2) illustrates the process of learning skip counting through the developed app.

From the flowchart (Figure 2), the proposed number is given as follows:

*N* = Σ(*V*_0_, ..., *V_n_*) **(13)**

where *N* is the sum of the expected value of coins and Σ(*V*_0_, ..., *V_n_*) is the sum of the current value of coins in the target space plus the value of the dropped coin *V_n_*.

Σ(*V*_0_, ..., *V_n_*) = *V*_0_ + *V*_1_, ^…^, + *V_n_* **(14)**

### Find Existing Interfaces

In this step, we identified existing interfaces such as iConverse [35], SPEAK all! [36], and 123 number [61] to build ideas into the system as much as practically possible prior to making it as a reference to build the actual system. These apps were installed on smartphones and tablets and given to the children to observe their ability to use these interfaces. The results from this observation were closer to those from our previous study, in which children with ASD were able to use digital gadgets when the apps installed are easy for them to use [5]. The same methodology of observation and FGDs was used in data collection of that study.

This step helped researchers to design a good user interface which depends on how often the users will be using the system compared with how often they will be using systems that they already know [58].

### Rough Out the Design

**Figure 3:**
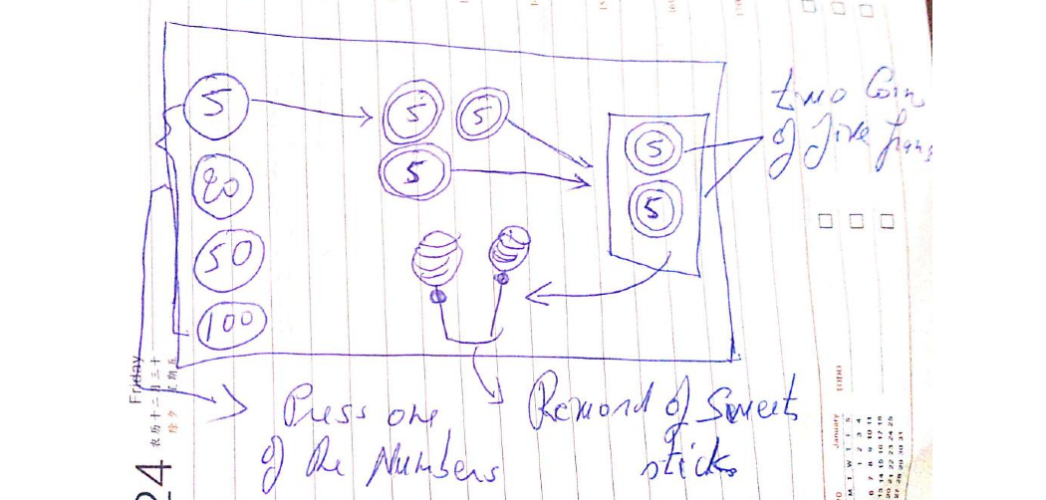
Rough out the design.

First, the preliminary (rough) description of the design is penciled on a paper. In this stage, the researchers and education practitioners had in-depth discussions on the features the system should have before building a prototype so that it can be tested out with the end users. Figure 3 presents an example of the design that simulates the intended interface.

As can be seen in the figure, a child with ASD presses and drags 1 image of a coin and drops it into the designed space at the right-hand side. The system will count the number of currencies the child puts into the space to present the reward. The child can repeat the same action as much as s/he can.

### Analyze User Interaction

Before designing the mock-up or prototype, we first analyzed how the users can interact with the interface that has been roughed out while performing specific tasks. At this stage, researchers identified areas where the users might make mistakes, such as pressing the wrong icon of coins or trying to go back. The analysis of the temporary interface was also supported by results from previous steps and experiences of study participants.

To use the developed app, children with ASD level 3 will get support from their teachers, parents, or family members who live with them.

### Create a Prototype

The prototype of this app was designed using Adobe Captivate version 19.0 [62], which has features to design a responsive interface and is compatible with both Android [63], the common platform installed in smartphones, and iOS, installed on Apple devices [64].

**Figure 4:**
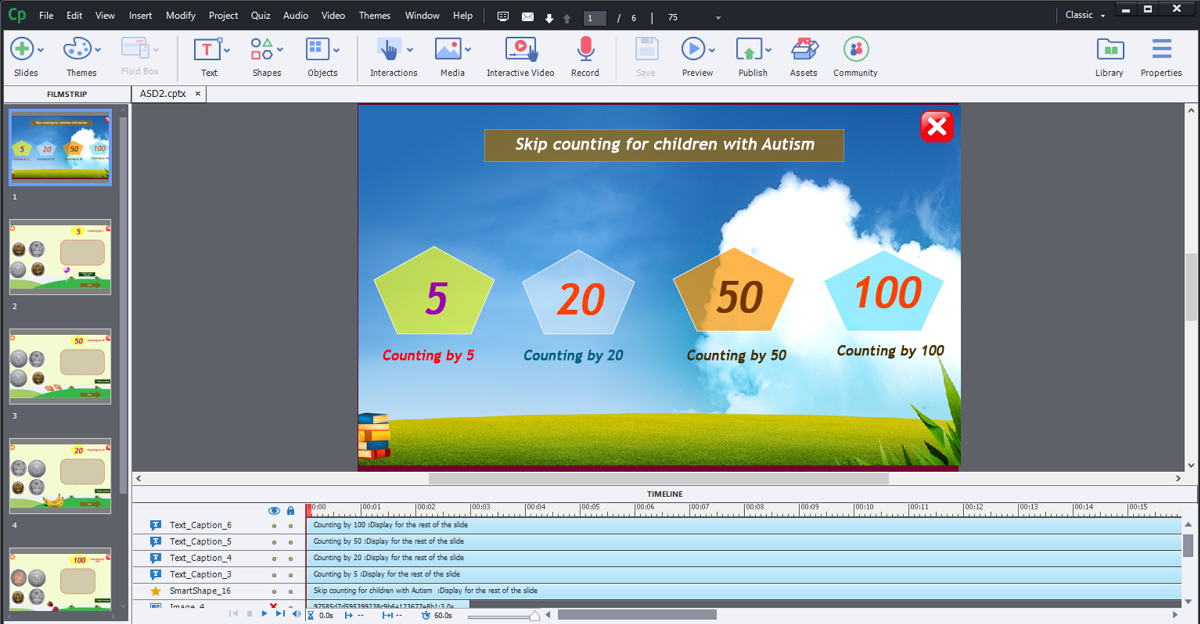
Adobe Captivate interface.

This prototype was made only to find flaws in the system and ways to improve them in terms of functionality and usability. The Adobe Captivate interface (Figure 4) shows the template used to design the user interface of the mobile app.

### Test Design With Users

Afterward, the prototype built was tested by chosen users at Autisme Rwanda to bridge the gap that might still exist. This stage helped researchers to further improve the system to suit children with ASD in Rwanda. The researchers were then able to analyze the list of features that may need to be improved. After testing the interface with the children, FGD was completed with educators at the Autisme Rwanda Center to get more information on the interface design.

The children were identified randomly from the class, regardless of their subject and the economic status of their parents. The gender did not influence children’s interaction during the test.

Both teachers and parents helped researchers in the recruitment of children that tested the app and continuously supported us in monitoring the changes in the behavior of these children. Parents of children who are not in school supported us by monitoring their children when they used the app at their homes during the test period. Every child that participated in this study was given a tablet on which the app was installed. The children used the app for a 30-day period.

### Iterate

Based on the test phase results, the participants (teachers and parents) reported problems that need to be accounted for, such as changes in the background colors of the app, images, and feedback messages. The researchers re-designed the app to make the recommended changes and reverified it with the participants. The design of the interface was revised 2 times.

### Build the Design

The final step in the development of this app before it may be commercialized is building the product. We built the app after solving the problems identified in the previous steps and FGDs. Continuous testing was performed to modify the interface according to the needs of children with ASD whose behavior may vary from one another.

The developed app was installed on smartphones and tablets that will be used in the next study to evaluate the impact of this app on children who will be using it.

### Ethics

With respect of research ethics, a letter of consent (Multimedia Appendix 3) was signed by each teacher and parent of children with ASD who participated in the study. Participation in the study was entirely voluntary and no special reward was given to the participants at the end of sessions as an acknowledgment of their time. Ethical clearance (Multimedia Appendix 4) was obtained from the Collegial Research Ethical Committee of the University of Rwanda – College of Education. The collaboration letter between the principal investigator (TN) and Autisme Rwanda was signed before the start of the study (Multimedia Appendix 5). The collected data are stored in a confidential and secure place on a physical memory drive.

## Results

### Identified Themes

A total of 10 themes emerged while developing this mobile app that responds to the needs of children with ASD in order to improve their numeracy skills (basic mathematics). The themes are related to (1) teaching and learning numeracy for children with ASD, (2) planning and development of a mobile app for a person with ASD, (3) testing a mobile app, (4) strength of the developed app against the existing ones, (5) behavioral maintenance and relapse prevention, (6) possibilities to integrate the mobile app into existing curriculum, (7) data protection for users, (8) social implications, (9) challenges in Rwanda, and (10) focus on future.

### Teaching and Learning Numeracy for Children With ASD

This theme elucidates models and techniques used to support learning numeracy for children with ASD. Teachers understood the application of the applied behavior analysis model in a classroom environment and were trying to implement some of its strategies to motivate children with ASD to stay focused. This was supported by a common statement from teachers which says:

Learners with Autism need rewards like giving them a pen or a toy to encourage them to study. We use available tools in the school like pens or rulers to motivate them to learn.

This succeeded when the teachers considered the needs of each child, as they are different across the spectrum. This is supported by an international expert who replied to the question posted on a public forum:

You must consider the needs of each child, as everybody on the spectrum is different. Some people on the Autism Spectrum might find it difficult even to do simple math problems, while others may find even complicated math problems too easy. Some may need more help with the X and Y values, while others may need more help with counting money.

This statement suggests that individuals with ASD do not learn the same way and thus the methods for teaching them need to be different. In the public forum, a person with Asperger syndrome said

Well, the first flaw in your question is that you assume we all learn the same. We don’t. However, if you take the approach that all individuals have different learning styles, and you try to incorporate that into your app, you might find a modicum of success.

Changes in methodologies and integration of digital tools that respond to the individual needs of a child with ASD can help teachers teach numeracy skills for these children.

### Planning and Development of a Mobile App for a Person With ASD

This theme explains the approach to follow when planning and developing a mobile app for children with ASD. It is important to familiarize the environment in which children with ASD live. This is confirmed by the period for which a researcher spent time in the school caring for children with ASD and the collaboration letter signed by both the manager of the center and the principal investigator (TN) of this research. While planning the development of an app that helps children with ASD learn, the involved collaborators sat together and examined the behavior of children with ASD under different conditions.

Equations 1-4 show that to design an interface for children with ASD, the designer considered (1) the senses of the children such as vision, hearing, and tactile; (2) cognitive level of a child to perform a given task; and (3) the ability to exercise motor functions to perform a task.

A list of user’s requirements (Table 1) was collected from participants when planning the development of the mobile app.

**Table 1:** List of user’s requirements.

Requirements/Difficulties	Requirements	Methods for gathering requirements
Senses (Sn)	For each action, there must be a voice associated with it. Use clear images of coins from the Rwandan currency system. Number of images of coins should be ≤5. The size of icons/coins must be big enough to enable the precisions of children when dragging them to the right place. Ignore the background sounds.	Group interviews, observation, literature review, and online open discussion.
Cognitive functions (Cg)	Use of a soft sound that can attract children to use the mobile app. Facilitate direct feedback using an image of the product that is liked by children, such as doughnuts, bananas, and candy. Reduce background colors that can distract children. Better to use 1 background image whose existence is known to children. Use a clear image that tells the user to return to the home screen or to close the app.	Group interviews, observation, literature review, and online open discussion.
Motor function (Mp)	The user interface needs to be responsive to devices with high resolutions such as tablets. Allow the user to repeat actions until the dragged object reaches the target. Choose the app to be in a landscape position.	Group interviews, observation, and literature review.

Equations 1, 2, 3, and 5 were also supported by a common response from the global practitioners who replied to the questions posted on the forum that the app design should take advantage of the interaction strengths by making the accessibility limited to skill instruction and improve information processing by being both engaging and enjoyable.

The participants suggested 3 points to consider when designing the interface for children with ASD: (1) graphic design guidelines, (2) user interface design, and (3) success recognition and messages.

### Graphic Design Guidelines

To design the graphical interface, we followed the recommendations by Hussain et al [65] who suggested the interface, number of pictures, size of the screen, icons, colors, and content to ensure the comfort of children with ASD when they use mobile apps. The graphic interface is made up of 2 main menus: (1) home and (2) exit.

**Figure 5:**
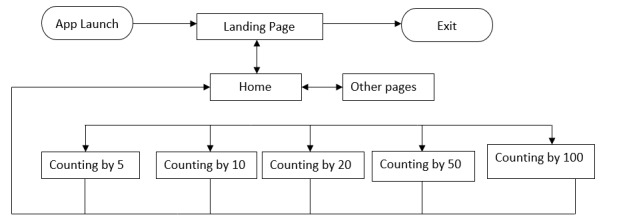
Design flow of the app.

The design flow (Figure 5) illustrates the app’s structure that facilitates users to navigate.

Figure 5 shows that the home menu is the starting point when the app is opened while the exit icon is used only to close it. Equation 5 defined a software app as a set of functions that are associated with user interface. Home menu, exit, and images that illustrate numbers to count are the elements of the developed mobile app’s interface. Equation 6 substituted the functionalities with tasks that users are expected to do within a software app via an interface. In this app, a child with ASD presses one of the figures to start counting numbers.

Equation 7 shows that an interface (*I*) is a set of user interface patterns (*Uip*) that facilitate users to interact with the system. From the home menu, a user can navigate through the system by touching 1 out of 5 icons: (1) counting by 5, (2) counting by 10, (3) counting by 20, (4) counting by 50, and (5) counting by 100. Equation 8 shows that a child with ASD who presents different characteristics presses one of these icons to start counting to achieve a certain goal.

Equation 9 demonstrates that under each submenu, a user counts Rwandan francs coins [66] that correspond to the number he or she heard from the voice (Equations 1-4).

To perform a task presented in Equations 10 and 11, a child with ASD drags at least one coin to a provided space to get the feedback (eg, candy, banana, strawberries, and others). The feedback for each action is the interfaces proposed in Equation 12.

### User Interface Design

The user interface is mainly divided into 2 categories based on menus specified in Figure 5: (1) landing page and (2) other pages.

#### Landing Page

**Figure 6:**
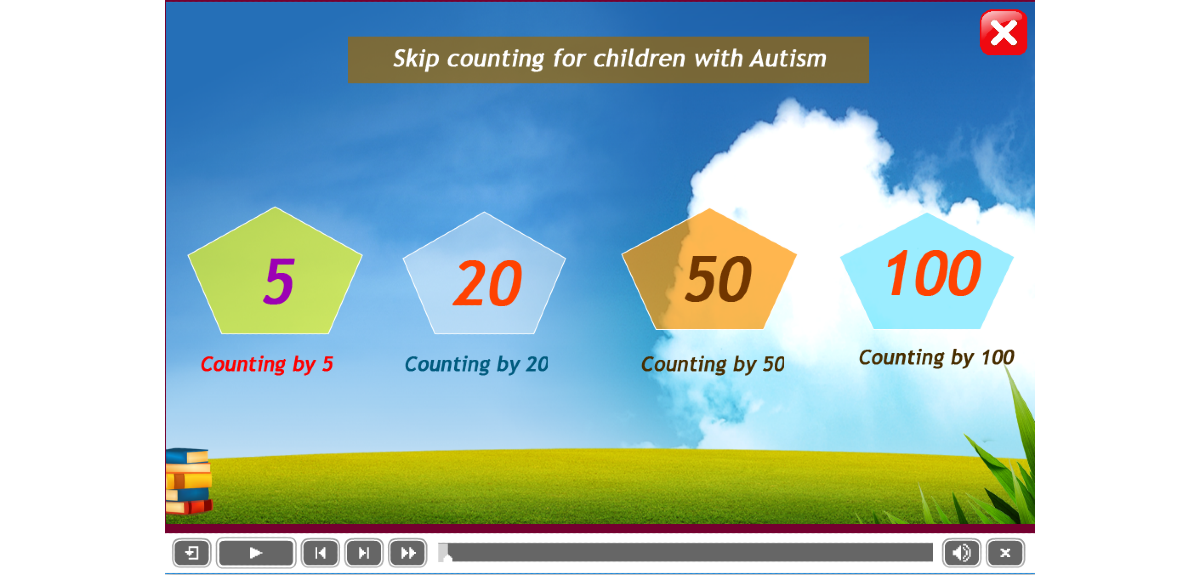
The landing page of the app.

After launching the app, the landing page shows all categories of skip counting numbers. The images of currency were not put on the home page to prevent the loss of concentration of children as recommended in Equation 3. Figure 6 shows the design of the landing page of the app, which presents 4 options, among which a user can press one to initiate the operation (Equations 5 and 6).

#### Other Pages

**Figure 7:**
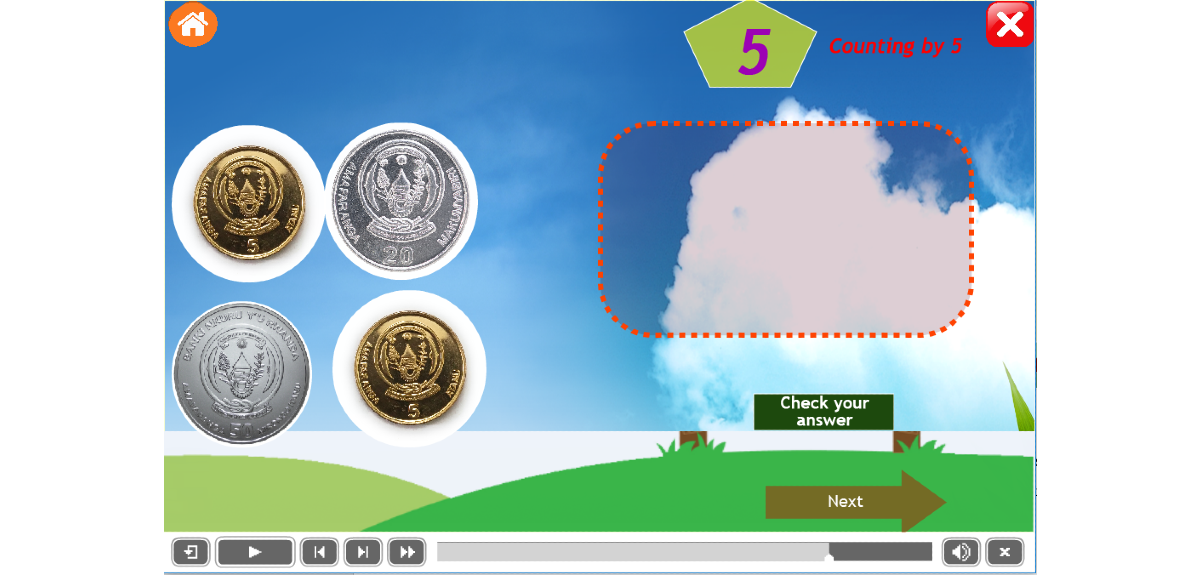
Counting by 5 interface.

**Figure 8:**
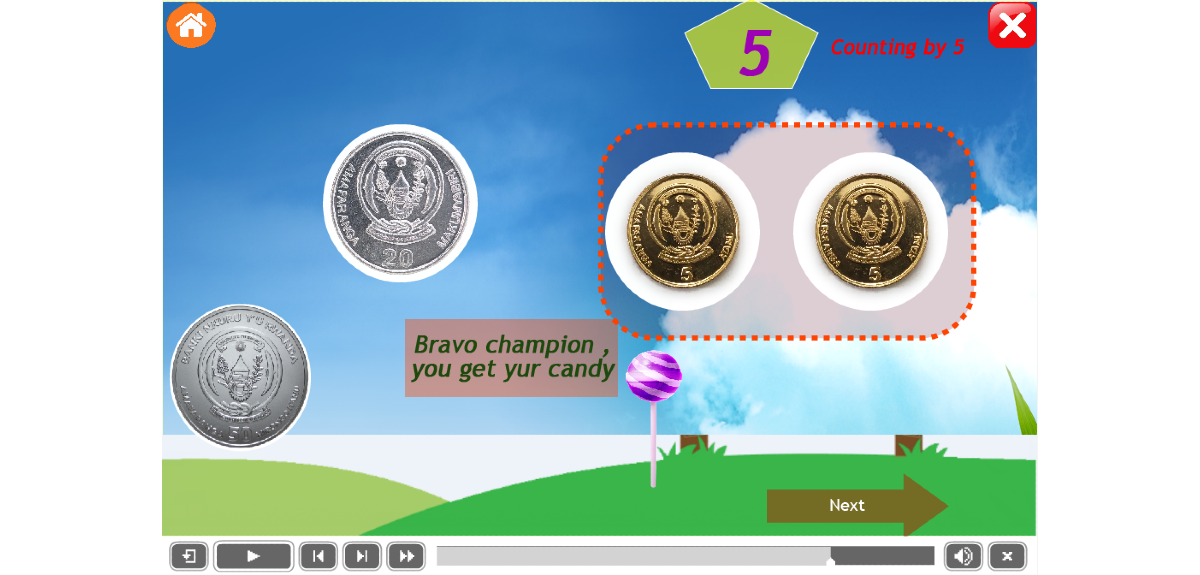
The interface of counting by 5 after dragging 2 coins of 5 francs.

Other pages become viewable after pressing an icon from the landing page as indicated in Equation 9. Each task is associated with a specific use interface (Equation 6). The interface contains a set of coins that are used in the Rwandan currency system, namely, 5 francs, 10 francs, 20 francs, 50 francs, and 100 francs [65]. The child is asked to drag coins one by one to the destination place to get a reward. Figures 7 and 8 show examples of the pages of counting by 5 before and after an operation, respectively. A child identifies coins of 5 francs and drags them into the box at the right-hand side and then after a successful operation s/he is given a reward of candy (Equations 10 and 11).

A child is asked to drag and drop coins of 5 francs to get a candy (Figure 7). Equation 13 shows that the current value is set to 0 initially, with values added after dropping each coin, as presented in Equation 14.

Using an example of counting by 5, the value of Equation 14 is as follows:

*N*=*V*_0_+*V*_1_+^…^+ *V_n_*

*V*_0_=0, *V*_1_=5, *V*_2_=5

*N*=0+5+5

*N*=10

Thus, the price of 1 candy is 10 Rwandan francs. Figure 8 shows the gift of candy offered to a child after a successful operation. In each step, the child with ASD has an interface containing the image, text, and audio (Equation 9).

### Success Recognition and Messages

**Figure 9:**
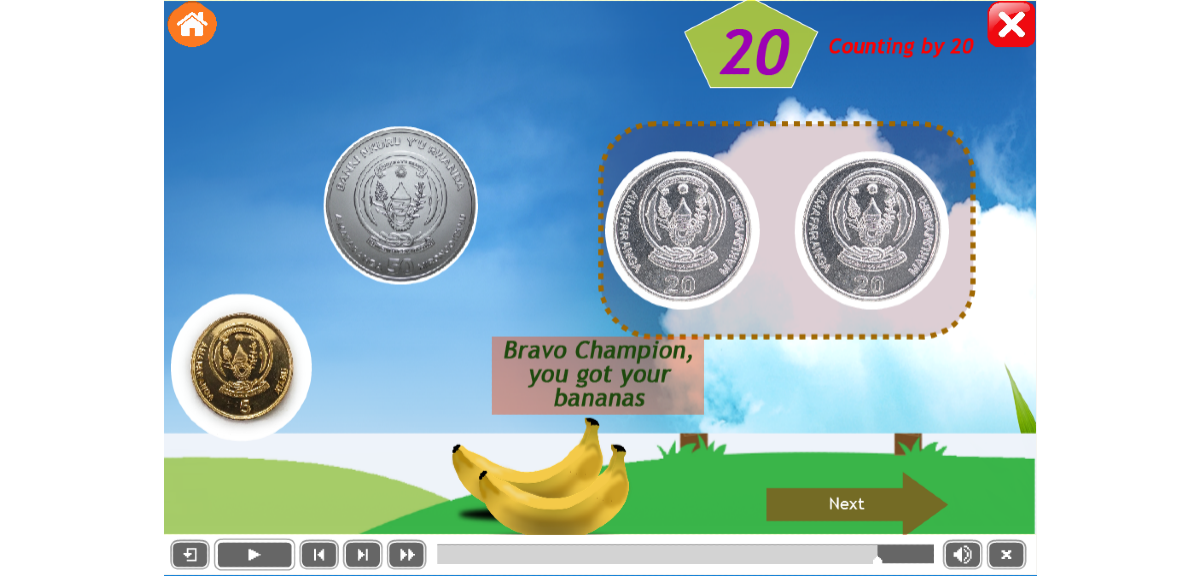
The reward of bananas after dragging 2 coins of 20 francs.

**Figure 10:**
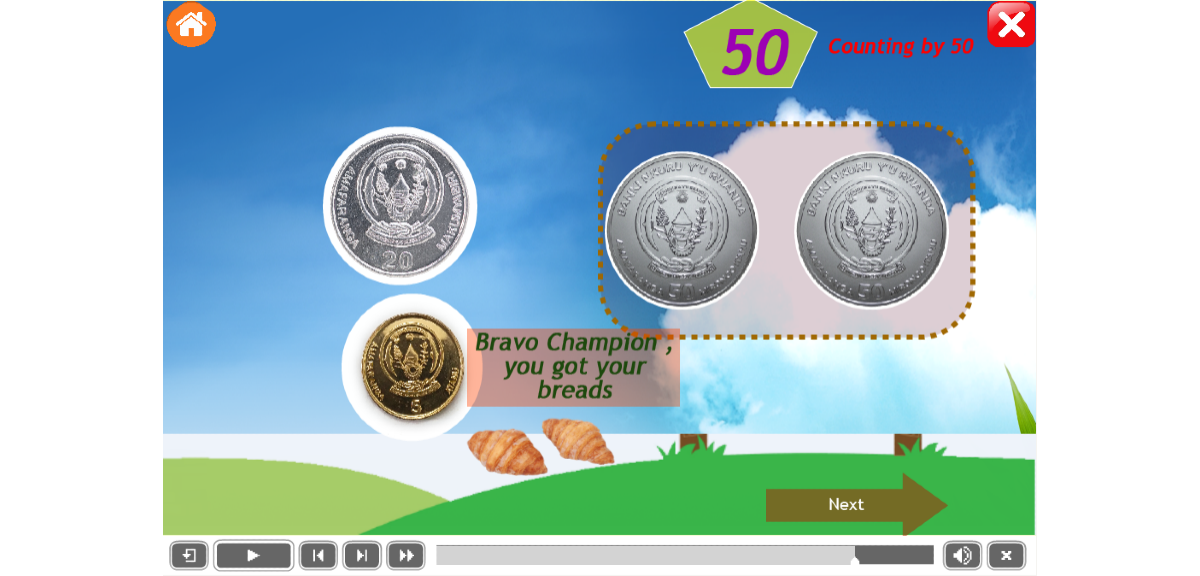
The reward of doughnuts after dragging 2 coins of 50 francs.

**Figure 11:**
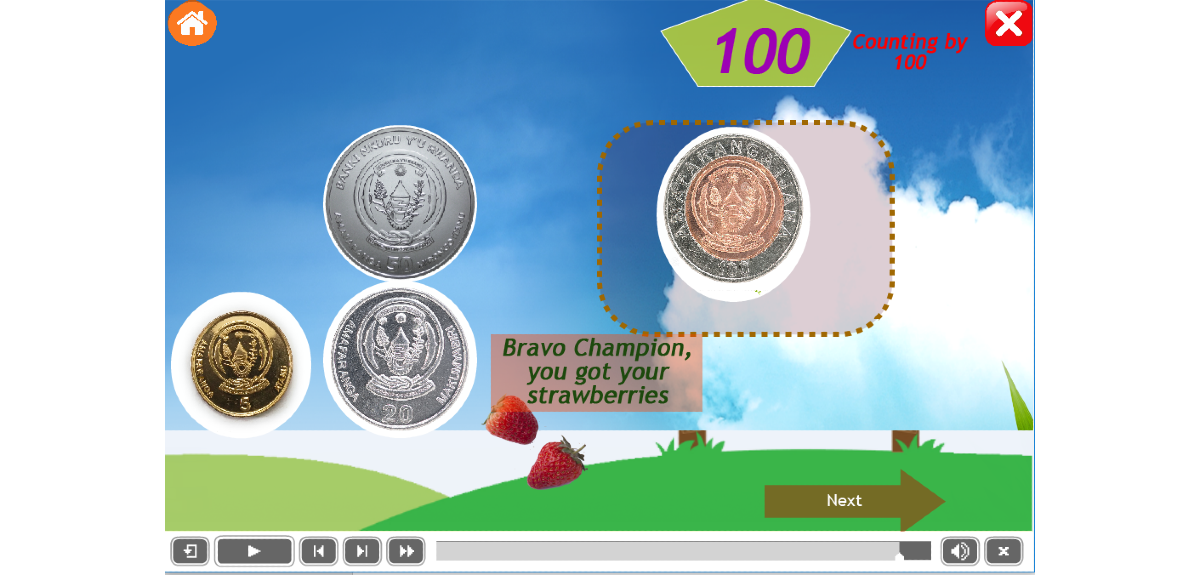
The reward of strawberries after dragging 2 coins of 100 francs.

To encourage children with ASD, it was recommended to award a special gift at every step of the operation. This mobile app provides different rewards that are common in their surrounding environment, such as doughnuts, bananas, and candies. Some example are as follows: the reward of bananas after dragging 2 coins of 20 francs (Figure 9), the reward of doughnuts after dragging 2 coins of 50 francs (Figure 10), and the reward of strawberries after dragging 2 coins of 100 francs (Figure 11).

Children with ASD were interested in the recognition message and image of the product which was offered to them after successfully completing an operation. This was supported by a teacher who said:

Children are most excited with the sound of congratulations after they succeed to count coins. As an educator, I found that this application can help much in my teaching activities when it will be improved to cover the whole curriculum.

The integration of visual and audio features in the ICT-enabled technologies can improve the attention of children with ASD when they are learning. In this app, every action is associated with a sound that informs the child what s/he is expected to do to get a reward.

Figure 10 shows a reward of 2 doughnuts after a child successfully counted 2 coins of 50 Rwandan francs. Counting 2 coins makes the child happy because of the reward—2 doughnuts. Continuing the exercises in this app stimulates children to be engaged in using the local currency to buy basic needs.

The changes in the product of reward encourage children to continue using the mobile app to find the next message and product. Figure 11 shows that the child succeeded in dragging 1 coin of 100 Rwandan francs and got a reward of 2 strawberries.

The children who use the developed mobile app get a reward after successfully counting the number of coins that are equal to the cost of the product (reward) set in the system. The children get a message to try again when s/he dragged the wrong coin.

### Testing a Mobile App

This theme demonstrates the result of testing the developed app. A total of 35 children in the Autisme Rwanda tested the app, which was monitored by the teachers in the center. Five children who are not in the center were visited at their homes. Table 2 presents an overview of staff and children at the center.

**Table 2:** Status of Autisme Rwanda in 2020.

Information on children and staff			Value
**Children, n**			
	Male	38	
	Female	7	
	Total	45	
Children age (years), range			2-12
**Staff, n**			
	Teachers	12	
	Teachers’ assistants	8	
	Technicians and hygienic personnel	16	
	Total	36	

An observation of the children using the developed app demonstrated its success, as it answered to their individual needs.

Findings from the test showed that it is crucial to customize the app in different forms, but with the same goal, so as to cater to each child’s behavior.

This was supported by a comment by a participant on the Quora platform who responded to the question regarding the background color to be used in the app:

That's totally up to the child. Many of them don't show any special indication of liking certain colors. When my autistic son was young, he liked orange, but after about the age of seven, he wore nothing but black. He's 36 years old and still wears nothing but black. Occasionally we can talk him into charcoal, but it doesn't last long.

The development of apps for children with ASD is a long process to accompany an individual in his/her life. It is therefore crucial to always change the content and format of the app to respond to the behavior of each child with ASD. These changes are also supported by another parent from the Quora forum who said

My son loves very intense, bright colors. But that doesn't mean everyone on the spectrum would. They are all individuals.

While suggestions were reported by some users, many found the app promising to improve the learning of children with ASD in Rwanda. This was supported by a parent who said,

What I can see is that by using this application, my kid will gradually increase the knowledge in counting money and I hope it will help him to use that knowledge in the society even though he is not enrolled in the school.

Using ICT tools, children with ASD learn more skills, which they can employ in their daily lives without being at school.

Children using the app showed more interest in continuing to use with the app, as they are familiar with the images of coins used in the app, which were similar to the ones they use in their families. This was supported by one parent who said,

My son was very interested in using this application because he always sees the coins we use at home. I believe within the time; my child will be able to count money.

Children with ASD can learn counting from the electronic device when the app is contextualized with the environment.

Table 3 shows the phases in which 55 participants participated during the design, development, test, and evaluation of the mobile app.

**Table 3:** The number of participants.

Participants	Design and evaluation, n	Development, n	Test, n	Total, n
Teacher	5	0	0	5
Parents	10	0	0	10
Children with ASD enrolled in a school	0	0	35	35
Children with ASD not enrolled in school	0	0	5	5
Total	15	0	40	55

### Strength of the Developed App Against the Existing Apps

This theme confirmed the positive effect of the app on children with ASD in their learning of numeracy skills. However, the result from the observation of children with ASD using SPEAK all! [36] and 123 number [61] indicated that it was difficult to adapt the existing interface to the needs of children with ASD when they were developed in a context different from what they are currently living in.

This was supported by all teachers who participated in this study. One of the teachers said,

Normally these children face the problem of communication in general. It is very difficult for them to learn new content which is in the foreign language and the content is not adapted to the local environment.

Many teachers and parents suggested re-creation of content adapted to the existing environment to enable children with ASD to learn from the objects they are familiar with.

The participants found the developed mobile app to have more impact, compared with other apps, on the learning of children with ASD in Rwanda.

This was supported by a common statement from parents:

This application will have a positive impact on the learning of our children because the coins used in the application are those we use in our daily life.

Despite this positive statement, participants suggested the need to have the app in the local language. Children with ASD have difficulties in verbal communication. Thus, it is crucial to use their mother tongue to enable them to learn new skills.

### Behavioral Maintenance and Relapse Prevention

Children with ASD tend to lose focus when communicating with others. This mostly happens due to distraction, which is commonly noted in these children in an inclusive environment. The instant motivational feedback when a child completes the operation makes him/her focus on using the app.

This is supported by an autism consultant who replied to a question posted on the Quora forum:

Just because they might not look at you does not mean that they are not paying attention or focusing. Is their ability to focus compromised by the sensory onslaught that is going on in the room?

The participants also suggested the consideration of different factors such as level of noise in the room, smells, and movement that can make children with ASD lose their focus. These factors can be also associated with the health conditions as reported by the same consultant.

Some autistic people have difficulty with interception, understanding messages from our body. The child might have a headache, earache, stomachache, etc. but not be able to let you know. Or, this might be a constant state for them, again, making it harder to focus.

The parents who participated in the study suggested giving time to the children when they are not following the instructions.

The children with ASD generally do not follow all the instructions we are giving them, but we are surprised to see them practicing what we were expected from them to do when they follow the instructions, and we hope with ICT can help better to improve their level of skills.

A common statement from parents

The provision of educational mobile apps that provide instant feedback at every step can help children with ASD stay focused and gradually improve their level of skills in different subjects.

### Possibilities to Integrate the Mobile App Into Existing Curriculum

Teachers who participated in this study said that children with ASD enjoyed using digital devices that have audio and visual features. Thus, it is recommended to provide digital devices in the class with content related to the intended learning outcomes.

ICT can bring a positive impact when they are used in class, but it is better to select applications that can respond to the planned objective.

A common statement from teachers

The results from the test phase of the developed app were promising for its use in one section of the mathematics syllabus in a competency-based curriculum.

I found that the level of children with ASD in my class has been improved after using this application. However, it takes much time to learn from it compared to normal students.

Learning a new subject for children with ASD takes longer than that for normal students. Therefore, the teachers suggested additional time to the time planned in the curriculum for normal students.

Response from parents proved that it is possible to learn basic mathematics outside the classroom by using ICT-enabled assistive technologies.

my kid is now trying to count money after using this mobile application. But it took time to learn it, it requires more time to expose the child to the application and play with it.

Mother of a child with ASD

Changes in the structure of the curriculum and integration of ICT in every section of the syllabus are recommended to include children with ASD in the normal educational system. This succeeds when the changes in methods of teaching children are also adopted.

An autistic child can learn anything that a neurotypical child can. The challenge is in the different approach to teaching (with careful consideration to the different way their brain/mind works) (e.g. using pictures rather than words to explain concepts, or using tactile feeling to aid memory/understanding), and the learning environment is accepting of and accommodating their needs.

Response from Adult diagnosed on the autism spectrum from Quora forum

The adaptation of content to children with ASD is possible when the use of text in teaching is reduced and the ICT is empowered with audio–visual features.

### Data Protection for Users

Privacy of information about users of mobile apps remains critical among participants.

The participants suggested not to expose any information about children with ASD. This information includes their images and information about their families. This policy was also supported by the school management which prohibited the use of children’s photograph within the app.

Parents suggested disabling the camera when children are using the mobile app and preventing the app from taking any other information from children when using it.

Usually in the community, there is a misunderstanding of the cause of the ASD. We accept our children, but we don’t want to share their images outside unless they are at school.

A common statement from parents

This is also supported by a teacher who said,

I remember when I was doing my academic research, one parent told me that many people are coming to visit him to do marketing of his child for business purposes. Now I cannot not allow anyone to take a picture of my child.

Although the use of ICT suggests the possibility of improving the learning ability of children with ASD, some features such as pervasive monitoring, facial recognition, biometrics, and blanket data retention practices are not recommended to be used in the educational app for children with ASD.

### Social Implications

This theme illustrates the social benefits and challenges associated with the use of the mobile app. Children with ASD have limited social communication skills, which is a major barrier to their learning. Thus, the participants expected the mobile app to improve the social relationship among children with ASD and the community.

I believe that the continuous use of mobile applications that are designed by considering this environment can also make children cope with the environment like going to shop, bringing some products and others.

A father of a child with ASD

Children with ASD can benefit from the practical knowledge of using money, thereby improving their relationship and communication with economic dealers.

However, some participants also reported a negative effect of the use of a mobile app for children with ASD that can affect their lives. They suggested time limit to restrict the continuous use of the app by children, which will also allow them to learn from other normal persons while not solely depending on the app for learning.

Using the mobile application is helpful to our children, however allowing them to learn only from the application can make them addicted with technology.

A common statement from teachers

Thus, mixing the technology with traditional educational interventions for children with ASD can bring about a positive impact on the life of these children and improve their social living with the community.

### Challenges in Rwanda

Changes in behavior and feelings among children with ASD may be a barrier to the development of ICT tools enabled with assistive technologies in Rwanda. The response from the Quora forum about the colors the children with ASD like indicates that their color preference changes with their age.

That's totally up to the child. Many of them don't show any special indication of liking certain colors. When my autistic son was young, he liked orange, but after about the age of seven he wore nothing but black. He's 36 years old and still wears nothing but black. Occasionally we can talk him into charcoal, but it doesn't last long.

Teachers who participated in this study suggested a continuous change of features in app depending on the behavior of a child.

The developed application can be helpful when children have a common preference. As we see it will succeed if the designer of the system can continually update it depending on the changes of behavior of our children.

A common statement from teachers

The development of a mobile app for children with ASD requires continuous iteration in the design process. However, the insufficient number of ICT technicians and qualified teachers available to participate in the design and development of a mobile app that responds to the needs of children with ASD remains a challenge in Rwanda.

Participants also reported difficulties in accessing the app because it can only be used on tablets or smartphones. This makes the mobile app inaccessible for children from families with a less privileged background. The provision of low-cost devices to families with low income is recommended by the participants.

### Focus on Future

The last theme is to identify the views and recommendations from participants to be considered in the future when planning and developing mobile apps for children with ASD. The findings of this study suggested the importance of developing a family-centered process that requires parents to support their children. In this study, most teachers suggested a collaboration between software developers to develop apps that respond to the needs of the children.

As we see, the design of the application for these children is a long process. We suggest building a good collaboration with application developers to make sure that the application can respond to the needs of the children.

A common statement form teachers

Developing a software app involves collaboration with the stakeholders in every step of development. However, if the users are persons with mental disorders such as ASD, this requires strong participation of persons who live with them. The development of a mobile app for the whole competency-based curriculum is also recommended by teachers.

The parents reported the use of the mobile app to support the transition of children from childhood to adult life if it is continuously updated according to the level of children and the changes in their behavior.

The behavior of our children is changed within time. We suggest a continuous change in application to accompany the children in their development till adulthood.

A common statement from parents

Using digital technologies supports social inclusion of children with ASD when these tools are systematically updated and meet the environmental context. Succeeding in the development and use of mobile apps for children with ASD requires a strong collaboration between educators, parents, and digital technology industries.

## Discussion

### Principal Findings

There has been continuous effort to increase community engagement in the development of learning tools for children with ASD. This study suggested continuous changes in the methodology used to teach children with ASD, and a systematic update of features in the developed app by considering the social norms, behavior, and privacy of these children. The responses collected from Quora [52] also suggested continuous changes in the features of the app so as to adapt it to the behavior of children with ASD during their different levels of development. This was supported by the study by Buteau-poulin et al [67], in which the authors recommended the regular update of information related to special education of children with disabilities that is available online. Participants from the Quora platform said that children can learn anything from digital devices even in cases where they tend not to look at the instructor during their class hours. This is possible only when the content meets the needs of the child and is structured around many different learning activities intended for real-world use [6,68].

We used coins and voices to keep the children focused. This was used as a reinforcement, as positive reinforcement has been utilized to keep children motivated during learning [7,69]. A study by Tzanakaki et al [70] reported that succeeding in teaching numeracy to children with ASD requires the use of various models that help students to stay focused. Moreover, children with ASD learn more easily from visual cues and videos promoted to enhance teaching and learning in an inclusive classroom [71,72]. Conoyer et al [73] evaluated different numeracy domains in the early grades such as oral counting, number recognition, touch count, missing number, and number relations. Hussain et al [65] recommended avoiding destructive voices and images that can make children lose focus. The interface designed in this study used 4 images of coins and each action is associated with a sound. Moreover, the success of ICT tools for education depends on the full participation of parents and educators of children with ASD [74]. Gamification of content [33] when teaching children with ASD is useful, as this made it possible to keep the children stay focused in this study; in this regard, having an interface that is more friendly and enjoyable is also important [56]. Many mobile apps were developed to support children with ASD [19], but some of them failed when used in different geographical contexts. To avoid this mistake, the interface designer in this study followed the guidelines prescribed previously [75] and the curriculum of basic education in Rwanda [44].

The inclusive curriculum, which aims at improving access and successful participation in the education of a person with disabilities, can bring a positive impact when ICT is fully integrated within the teaching and learning practices [76]. Results of this study show that by using ICT tools, children with ASD learn more skills commonly practiced in their daily lives without attending a school. However, it is difficult to follow the formal curriculum design when teaching children with ASD [15]. This is supported by different strategies [77], such as the development of competencies of teachers in teaching children with ASD before enrolling them into mainstream schools. The improvement of infrastructure, such as assistive technologies enabled with ICT, is reported to have a positive impact on facilitating the learning of children with ASD [78].

The study by Diener et al [79] reported that assistive technologies can help improve the inner skills hidden in children with ASD. The exposure of children with ASD to digital tools can bring about positive effects [80]. However, children with ASD are engaged only when more time is given to them to play with digital devices [5,81]. This approach is also supported by Song et al [82] who reported success in assisting persons with communication difficulties through ICT tools. Our results are no different. To sum up, children with ASD can benefit from app knowledge and utilize the knowledge gained to improve their communication in day-to-day economic activities.

Despite the advantages of using ICT tools in the education of children with ASD in Rwanda, there remain challenges of social implication when children with ASD are exposed to the digital tools for a long period [33,83]. However, parents and teachers are encouraged to supervise children with ASD to be able to manage challenging behavior(s) that may occur upon removal of the digital device [7]. Although the use of ICT tools in education continues to be encouraged to support learning of children with ASD, there is an increasing need for educational ICT tools such as mobile apps that support inclusion of children with ASD in the general education system [84]. By engaging the stakeholders to drive the development of the mobile app, we are addressing the needs of families and educators to improve the lives of children with ASD in the society.

### Strengths and Limitations

The researchers of this study intended to find an ICT solution that can support the current competency-based curriculum of basic education in the Rwandan education system.

This study has the following strengths:

- It reflects the experiences of teachers and parents who are in service to support children with ASD.
- It was conducted by experienced researchers in the field of education, information technology, special education, and ASD.
- The test was performed by children with ASD themselves while parents and teachers contributed to the improvement of the developed app.

This study followed the practical recommendations of Gowen et al [75], which helped researchers to familiarize with the autism community. These recommendations are (1) prestudy considerations, (2) recruitment of participants, (3) study visit considerations, and (4) poststudy considerations.

The rationale was that teachers can adapt to different new methods and tools that are innovative to improve the teaching and learning process. The researchers in this study selected skip counting as a learning method that can help children with ASD to improve their mathematical skills that are used in real life [73].

The study has the following limitations: (1) it explored only 1 topic among the 36 topics (3%) of planned contents of the syllabus that is supposed to help children learn basic numeracy at an early age and (2) there may be some long-term disadvantages experienced by the teachers due to constant changes and updates to the app (ie, technical and pedagogy challenges).

In future studies, all content corresponding to the existing syllabus should be explored and allow more time for participants to test the interface and take into consideration much of their views. A study on the impact of the developed app may help uncover both the long-term advantages and disadvantages that teachers may experience during the implementation of new curriculum. Another potential solution that should be further explored is the development of personalized ICT solutions for each subject planned in the competency-based curriculum that can respond to the needs of children with ASD.

### Conclusions

The ICT has promisingly paved its way into the field of teaching and treating children with ASD. Rapid advancement has been achieved by developing different ICT tools to improve the quality of life for children with ASD. However, the community plays a robust role in the planning, development, and evaluation of a mobile app for children with ASD. In this study, we focused on developing a user-friendly effective tool (a mobile app) that responds to the needs of children with ASD and eliminates the barriers of learning basic mathematical skills. The community and end users should play the pivotal role in the planning, development, and evaluation of any digital tools and techniques to ensure their acceptability and successful integration. This study may be the starting point for future studies that facilitate the successful integration of individuals with autism into the society of Rwanda by utilizing different digital tools in order to ensure their participation in different learning and economic activities.

## Appendix

Multimedia Appendix 1 Questions posed on Quora.

Multimedia Appendix 2 Interview guide.

Multimedia Appendix 3 Letter of consent/Participant consent form.

Multimedia Appendix 4 Ethical clearance.

Multimedia Appendix 5 Letter of collaboration.

## Abbreviations

ASD: autism spectrum disorder
CDC: Centers for Disease Control and Prevention
FGD: focus group discussion
ICT: information and communication technology
QNA: qualitative narrative analysis
RPIA: Rwanda Parent’s Initiative on Autism
