# Supplementary material for: Development of a Mobile App to Improve Numeracy Skills of Children With Autism Spectrum Disorder: Participatory Design and Usability Study
Source: JMIR Pediatr Parent. 2021 Aug 31;4(3):e21471. doi: 10.2196/21471 (PMC8441616; doi:10.2196/21471)
Supplement: Multimedia Appendix 1 [file pediatrics_v4i3e21471_app1.docx]

## **Appendix 1**

Question 1: What colors do children with autism-like the most when using a mobile application?

**(https://www.quora.com/What-colors-do-children-with-autism-like-the-most-when-using-a-mobile-application)**

Question 2: What are technical design of mobile app for person with Autism? [**https://www.quora.com/unanswered/What-are-technical-design-of-mobile-app-for-person-with-Autism**](https://www.quora.com/unanswered/What-are-technical-design-of-mobile-app-for-person-with-Autism)

Question 3: What are the key elements to consider when designing a mobile app to teach numeracy to children with autism?

[**https://www.quora.com/What-are-the-key-elements-to-consider-when-designing-a-mobile-app-to-teach-numeracy-to-children-with-autism**](https://www.quora.com/What-are-the-key-elements-to-consider-when-designing-a-mobile-app-to-teach-numeracy-to-children-with-autism)

Question 4: What are the key points to consider when teaching numeracy to children with autism to make them focus?

[**https://www.quora.com/What-are-the-key-elements-to-consider-when-designing-a-mobile-app-to-teach-numeracy-to-children-with-autism**](https://www.quora.com/What-are-the-key-elements-to-consider-when-designing-a-mobile-app-to-teach-numeracy-to-children-with-autism)

Question 5: Can a child with autism study numeracy?

[**https://www.quora.com/Can-a-child-with-autism-study-numeracy**](https://www.quora.com/Can-a-child-with-autism-study-numeracy)
