# Supplementary material for: Development of a Mobile App to Improve Numeracy Skills of Children With Autism Spectrum Disorder: Participatory Design and Usability Study
Source: JMIR Pediatr Parent. 2021 Aug 31;4(3):e21471. doi: 10.2196/21471 (PMC8441616; doi:10.2196/21471)
Supplement: Multimedia Appendix 2 [file pediatrics_v4i3e21471_app2.docx]

**Appendix 2.**

1. Describe your teaching method – how would it help pupils get to grips with Maths?
2. How do you plan to use technology in a typical Maths lesson?
3. How would you boost a pupil's attention when you are teaching math?
4. How do you find this interface to help the learning of children with cognitive disabilities like those who have Autism?
5. As I introduced, the goal of this research is to develop a mobile application to enable children with ASD to learn from the apps.
6. What are your suggestions to be done on the interface to meet the Rwandan Context?
7. What are the challenges you find to be addressed before using this interface?
8. What are elements can be included in the interface to help children with Autism to stay focus?
9. Teaching children with Autism in class we need reward actions for them. How do you reward the children in a class to encourage them in their learning?
10. What do you suggest focusing on in the future?
11. To close this discussion, do you have any other comments or suggestions that may contribute to this study?
