# Supplementary material for: Development of a Mobile App to Improve Numeracy Skills of Children With Autism Spectrum Disorder: Participatory Design and Usability Study
Source: JMIR Pediatr Parent. 2021 Aug 31;4(3):e21471. doi: 10.2196/21471 (PMC8441616; doi:10.2196/21471)
Supplement: Multimedia Appendix 3 [file pediatrics_v4i3e21471_app3.docx]

**Informed Consent Form for teachers and parents**

This informed consent form is for teachers of children participating in the research study titled: “**Development of a mobile application to improve numeracy skills of children with Autism Spectrum Disorders in Rwanda**”

The study is conducted by Théoneste Ntalindwa, Nduwingoma Mathias, Karangwa Evariste, Alphonse Uworwabayeho, Annette Uwineza (University of Rwanda, Rwanda), and Tanjir Rashid Soron (Neuro Developmental Disabilities Protection Trust, Bangladesh).

This Informed Consent Form has two parts:

Information Sheet (to share information about the study with you)

Certificate of Consent (for signatures if you agree that your child may participate)

**Part 1: Information Sheet**

**Introduction**

We are doing research to enable people with Autism Spectrum Disorders to be included in basic education system in Rwanda. In short, this study aiming at design, development and evaluation of a mobile application to improve learning basic mathematics for children with Autism in Rwanda.

In our research, we need to work with teachers and parents in the design and evalution of the mobile application. We will work with children with ASD in testing of how they can use the developed application.

To do this we ask you as a teacher / parent for permission. After reading about the study below, and if you agree, then the next thing we will do is ask you for their agreement as well, before the session.

There may be some words that you do not understand. In that case, please feel free to either contact us via e-mail or the principal at the school, who can send us all your questions via e-mail so we can answer it before you sign the certificate of consent.

**Voluntary participation**

You do not have to agree that you can participate in the study. You can choose to say no and any services that you receive at the school will not change. We know that the decision can be difficult. You can ask as many questions as you like, and we take the time to answer them via the principal. You do not have to answer any question or take part in the observation if you feel uncomfortable doing so.

**Procedure**

The study will be done at the **Autisme Rwanda.** You will participate in one or more sessions session of about 30 minutes in focus group discussion. The interviews will be recorded.

The questions that are likely to be asked during the focus group discussion will be related to the main research question: Is it possible to design a mobile application that helps children with ASD to learn skip counting using coins used in Rwandan currency?

**Benefits**

There will be no immediate and direct benefit to you or your school, but your participation is likely to help us find out more about how to develop a mobile application to be made more accessible and support inclusion of person with neuro-developmental disabilities like ASD in education system with others normal students. We hope that the results will help integration of ICT in your teaching profession and enabling your child to perform like others at the school as well as improving communication between you and your pupils.

**Reimbursements**

You will not be provided with any payment to take part in the research. However, the prototype of the application will be made available for free.

**Confidentiality**

We as researchers will not share information about you. However, because something out of the ordinary is being done through research in your community, it will draw attention. If you participate, you may be asked questions by other people in the community. We cannot guarantee confidentiality, but it is our belief that the nature of the research question is not very sensitive or personal.

**Sharing of Research Findings**

We will share what we have learnt with the participants and the principal. Nothing that your child will tell us during the sessions will be attributed to him/her by name. A written report will also be given to the participants, which they can share with their community. We will also publish the results in order that other interested people may learn from our research.

**Right to refuse or withdraw**

You may choose not to participate in this study. Choosing to participate or not will not affect either your own future at the school in any way. You still have all the benefits that would otherwise be available at this school. You stop participating in the observation at any time that you wish without either of you losing any of your rights.

**Who to Contact**

If you have any questions you may ask them now or later, even after the study. If you wish to ask questions, you may contact either the principal or Théoneste Ntalindwa, [ntatheos@yahoo.co.uk](mailto:ntatheos@yahoo.co.uk) , Telephone: 0788884594. Mathias Nduwingoma [ndumathias2001@yahoo.com](mailto:ndumathias2001@yahoo.com), Telephone: 0788897814. Evariste Karwanga [karangwa81@ymail.com](mailto:karangwa81@ymail.com), Telephone: 0785489767.
