# Supplementary figures and images for: Development of a Mobile App to Improve Numeracy Skills of Children With Autism Spectrum Disorder: Participatory Design and Usability Study
Source: JMIR Pediatr Parent. 2021 Aug 31;4(3):e21471. doi: 10.2196/21471 (PMC8441616; doi:10.2196/21471)

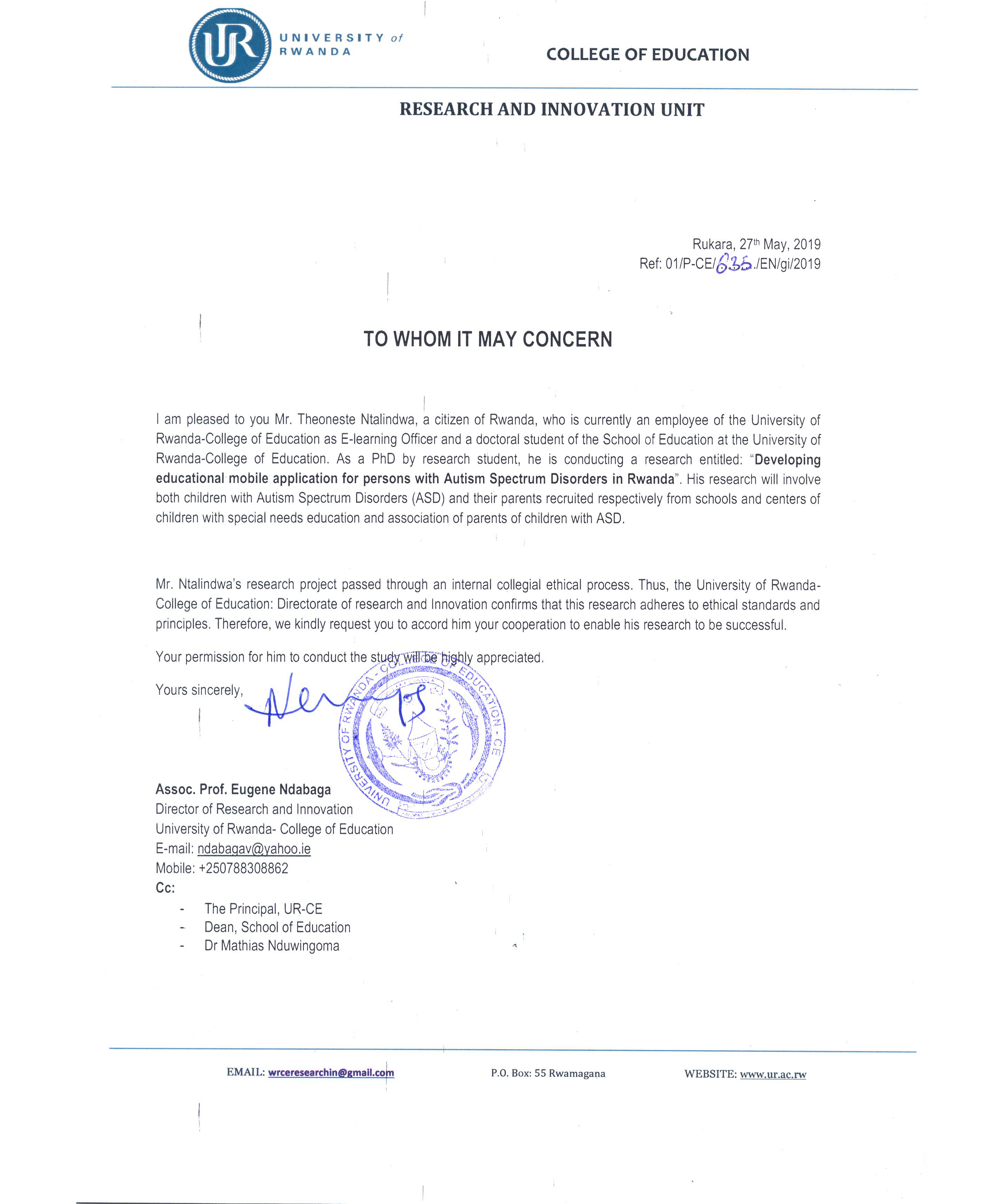

Supplement: Multimedia Appendix 4 [file pediatrics_v4i3e21471_app4.png]
