# Supplementary material for: Development of a Mobile App to Improve Numeracy Skills of Children With Autism Spectrum Disorder: Participatory Design and Usability Study
Source: JMIR Pediatr Parent. 2021 Aug 31;4(3):e21471. doi: 10.2196/21471 (PMC8441616; doi:10.2196/21471)
Supplement: Multimedia Appendix 5 [file pediatrics_v4i3e21471_app5.pdf]

autismerwanda@gmail.com/https://www.facebook.com/
www.autismerwanda.org
+250781521973,+ 250 782 414 220, +33677188974 Rosine Duquesne Kamagaju, Fondatrice

LETTER OF COLLABORATION

The purpose of this agreement is to define the reception conditions in the Autism Rwanda center and Théoneste Ntalindwa.

RECEPTION CENTER

NAME: AUTISM RWANDA

Represented by: ROSINE DUQUESNE KAMAGAJU

as General Manager

Address: KG 836 N ° 1 GISOZI

Telephone: +2507824141220

INTERN

Last Name and first name: Théoneste Ntalindwa

Date and place of birth: 08/17/1984 / Gicumbi

Nationality: Rwandan

Address: Kigali City - Gasabo-Jabana

EDUCATIONAL INSTITUTION OR TRAINING ORGANIZATION

NAME: University of Rwanda - College of Education

Represented by: Prof. George K. Njoroge

as: Principal

Address: principal.ce@ur.ac.rw

EDUCATION AND TRAINING

Nature of studies or training: Autism and management of autistic children

Duration: 4 years

autismerwanda@gmail.com/https://www.facebook.com/
www.autismerwanda.org
+250781521973,+ 250 782 414 220, +33677188974 Rosine Duquesne Kamagaju, Fondatrice

Degree prepared or qualification sought: Doctorate / PhD

Level reached (year of Bachelor, Master ...): Bachelors of Computer Sciences with Education, Kigali Institute of Education (KIE), 2009. Master of Science in Information Technolgy (MSc.IT), Univerity of Madras India, 2012

STUDY OBJECTIVES

The aim of the work is to ensure the practical application of the student's theoretical knowledge.

The student's missions within the center are as follows: Autism and understanding the appropriate care for persons with Autism within Autisme Rwanda

The educational objectives are:

- Develop a computer application to help learning for children with autism.

- Evaluation of the use of the application by children with autism

- Do advanced research to develop the intervention for children with Autism

Name, first name and position of the person in charge of the internship follow-up in the company:

Rosine Duquesne Kamagaju, Founder

STUDIES CARRIED OUT

The duration of study is (10) months. It takes place from (November 2019) to (September 2020). It will take place (place) (in case of multiple places, specify each of them and the corresponding dates)

EVALUATION

At the end of research:

autismerwanda@gmail.com/https://www.facebook.com/
www.autismerwanda.org
+250781521973,+ 250 782 414 220, +33677188974 Rosine Duquesne Kamagaju, Fondatrice

- Theoneste Ntalindwa is required to provide the Autism Rwanda center with a research report, a copy of which is communicated to the reception center.

- Théoneste Ntalindwa is required to mention in his research book the collaboration of Autism Rwanda in his research as well as the name of the legal representative.

SIGNATURES

Legal representative of the Autisme Rwanda signature and stamp

14.07.2020

Théoneste Ntalindwa
signature
